# Editorial: Esports: Playing Into the Future

**DOI:** 10.3389/fspor.2022.967560

**Published:** 2022-07-08

**Authors:** PI Joanne DiFrancisco-Donoghue, Gordon J. Schmidt, KIRILL Micallef Stafrace, Jerry R. Balentine

**Affiliations:** ^1^College of Osteopathic Medicine, New York Institute of Technology, New York, NY, United States; ^2^School of Health Professions, New York Institute of Technology, New York, NY, United States; ^3^Faculty of Medicine and Surgery, University of Malta, Msida, Malta

**Keywords:** esports, gaming, health, video games, players

## Why Esports?

The topic of esports spans multiple cross-disciplinary domains such as management, physical and mental wellbeing, performance, and psychology. Despite a year of extensive solicitations to research intensive institutions, we received only 11 articles, and eight were considered for publication. This demonstrates a paucity of data being conducted and published in esports. Competitive video gaming has a global appeal of more than 450 million viewers and has grown exponentially in the past few years with a surge resulting from the pandemic lockdowns (Esports the money game, 2022). The editors of this journal have reviewed the submissions and advocate for further scientific inquiry with an emphasis on data sharing on topics related to health, training, and nutritional aspects of esport athletes. McGee and Ho, confirm the injurious nature of esports especially related to tendinopathies associated with sustained wrist bending and twisting movements. A public health approach to combat injury for those who have sedentary lifestyles and unhealthy diets has been advocated by Ketelhut et al. Our intention is to highlight the current studies involving many more aspects of esport health and to bring greater prominence to the nascent field of esport research.

## Who are Esport Players?

Professional esport players are typically between the ages of 21 and 25 years, with a retirement age of 25 years (PMC, 2022). This is a time in human physiology when reflexes start to deteriorate, and chronic overuse musculoskeletal disorders start to limit performance and playing abilities (PMC, 2022).

These injuries are typically specific to the type of game played. Not all esport players use the same skill set, endure the same stress, or utilize the same muscles. Esports games vary on how player's skills are ranked and their expertise. Skill is determined by factors such as wins and losses and other in-game statistics. The specific in-game statistics, which influence in-game rank, vary for each game (PMC, 2022). There are various devices used that are game specific. For example, a mouse, keyboards, and console controllers, which all vary in sensitivity, weight (which affects force needed), the actions performed, and positions of the most common buttons or keys used (McGee and Ho). These variations can greatly impact the type of tendinopathies seen in players and how they should be individually evaluated. Overuse injuries and physical activity must be addressed with the focus being on both player performance and player health. The necessity for specific gaming equipment and game-specific regimens to decrease tendinopathies and other overuse injuries is critical for player health and lifespan in the future of esports research.

## Game Performance

There is a perception that people who play video games for long periods of time are physically inactive. Most of the literature conducted in gamers has been done on young males. What we have discovered is that these players, regardless of the amount of physical activity as recommended by the World Health Organization (WHO), sit for extended periods of time. Sleep deprivation and stress are harmful to one's health. Esport players rarely receive the same level of social support as traditional athletes at all levels. To advance to research that can show a cause and effect of interventions in esports and gaming, researchers must understand the self-reported behaviors of these players, their skill demand, the type of support they receive from within their social environment, their institutions, and direct from their teammates and coaches. In the following topic articles, Rudolf et al. investigate gamers' media consumption and wellbeing and Trotter et al. examine how gamers perceive their own social support and self-regulation. Additionally, there are very few studies that examine how to improve performance through various types of training. Neri et al. translate sport-specific training and demonstrate the benefit of individualized adaptive training in professional players over standardized play, whereas Boffel et al. demonstrate how character cosmetic microtransaction customization can influence performance.

## Healthcare Professionals and Seeking Help

The role health professionals play in the field of esports and competitive videogaming is still being defined. Building on the experience of traditional sports would imply that they would have an essential and central role in the management of the welfare of the athletes. Yet unlike traditional sports, esports players may lack the understanding and are therefore deprived of the benefit of screenings and counseling of healthcare professionals. As a result, additional data on gamers of various levels and ages are required. In this special edition of Frontiers in Sport and Active Living, one study illustrates how playing Minecraft can help middle-aged adults enhance their memory. Stark et al. Qualitative research and validated outcome measures are essential to establish a foundation for interventional strategies to protect the physical and mental wellbeing of players, while improving performance.

Studies included in this Research Topic identify areas in esports that relate to society, health, performance, and a call for continued research in all these domains, and others, among gamers of all levels and skills. The health of the gamer cannot be forsaken with such an exponential growth of esports.

## Author Contributions

All authors listed have made a substantial, direct, and intellectual contribution to the work and approved it for publication.

## Conflict of Interest

The authors declare that the research was conducted in the absence of any commercial or financial relationships that could be construed as a potential conflict of interest.

## Publisher's Note

All claims expressed in this article are solely those of the authors and do not necessarily represent those of their affiliated organizations, or those of the publisher, the editors and the reviewers. Any product that may be evaluated in this article, or claim that may be made by its manufacturer, is not guaranteed or endorsed by the publisher.
